# Sewer biofilms as drivers of greenhouse gas emissions in urban sewer systems: mechanisms and control strategies

**DOI:** 10.3389/fmicb.2026.1915507

**Published:** 2026-09-23

**Authors:** Linjun Zhang, Yongzhou Cheng, Yigao Xie, Min Guo, Shuchang Huang

**Affiliations:** 1 School of Intelligent Materials and New Energy, Institute of Carbon Neutral New Energy, Yuzhang Normal University, Nanchang, China; 2 School of Hydraulic and Ocean Engineering, Changsha University of Science and Technology, Changsha, China; 3 Environmental Engineering College, Jiangxi University of Science and Technology, Ganzhou, China

**Keywords:** greenhouse gas emissions, methane, mitigation strategies, sewer biofilm, urban sewer systems

## Abstract

Urban sewer systems function as active subsurface bioreactors, where dynamic biofilms drive significant methane (CH_4_) and nitrous oxide (N_2_O) emissions. Redox-stratified microenvironments within biofilms enable concurrent fermentation, methanogenesis, methane oxidation, and nitrogen transformations, linking hydraulic conditions to greenhouse gas generation. CH_4_ derives mainly from anaerobic micro-zones regulated by substrates and retention time, whereas N_2_O forms under micro-aerobic conditions via nitrification and incomplete denitrification. Current mitigation strategies, including physical cleaning, chemical dosing, and electron acceptor adjustments, often target isolated pollutants, neglecting inter-gas trade-offs. Methanotroph-mediated methane oxidation, including aerobic and nitrite-dependent anaerobic pathways, provides an additional biological route for mitigating sewer CH_4_ emissions. This review synthesizes biofilm-mediated emission mechanisms and control measures, advocating a shift from single-pollutant interventions toward precision, system-wide strategies. Integrating high-frequency monitoring, multi-omics, and dynamic modeling is essential to reconcile drainage functionality with climate goals and advance low-carbon, resilient urban wastewater infrastructure.

## Graphical Abstract

**Figure:**
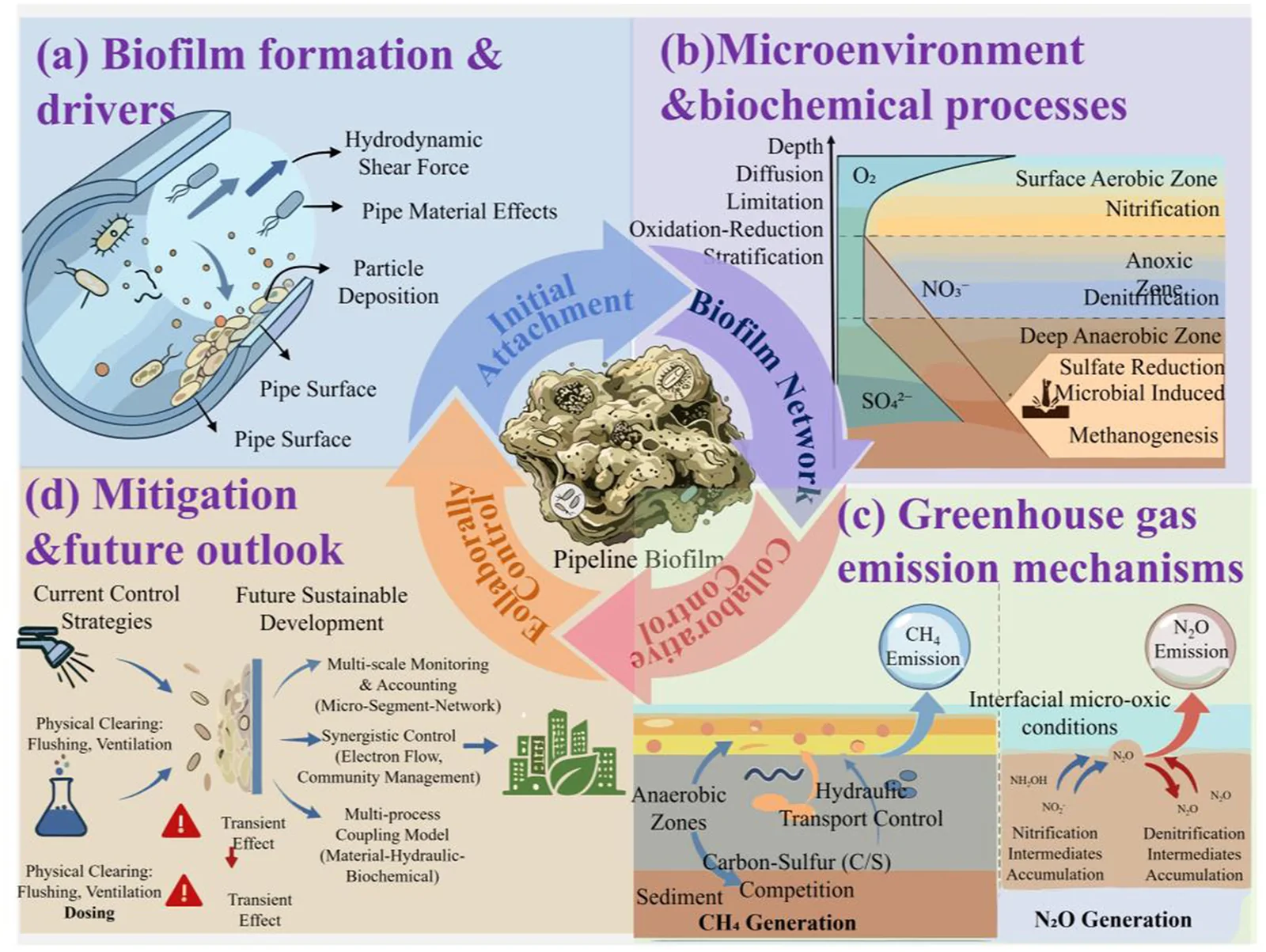

## 1 Introduction

Urban sewer systems, as a critical component of modern urban infrastructure, play a pivotal role in the safe and efficient conveyance of domestic and industrial wastewater (Price et al., 2026). Sewer networks function not merely as passive conduits but as active subsurface bioreactors, a conceptual shift driven by advances in understanding biofilm dynamics (Zeng and Sun, 2025). Within these environments, biofilms colonizing pipe interiors undergo continuous cycles of attachment, maturation, detachment, and re-dispersion (Ai et al., 2016; Zan et al., 2019). These biofilms provide a habitat and reactive interface for various microbial functional groups, such as sulfate-reducing bacteria, methanogenic archaea, and ammonia-oxidizing bacteria (Wang Z. et al., 2025). Consequently, sewer systems act as significant sources of greenhouse gases, particularly methane (CH_4_) and nitrous oxide (N_2_O) (Pei et al., 2025; Qi et al., 2026). Given the high global warming potentials of these compounds, sewer-derived emissions represent a non-negligible contribution to the carbon footprint of urban wastewater systems and warrant systematic integration into life-cycle assessment frameworks (Bai et al., 2026).

Sewer systems management has traditionally prioritized hydraulic performance and the prevention of physical blockages, while more recent efforts have begun to account for sewer-associated biochemical transformations and their contributions to carbon emissions (Chen J. et al., 2024). This evolving perspective reflects a growing consensus within the research community that sewer networks should no longer be regarded merely as passive conduits, but rather as active subsurface bioreactors (Wang X. M. et al., 2025). In response to these challenges, a range of control strategies has been developed and, in some contexts, implemented. These include physical cleaning techniques (Mohit et al., 2025), chemical interventions such as nitrate (Zuo Z. et al., 2024) and iron salts (Yan et al., 2023). Beyond suppressing methane production, methanotroph-mediated methane oxidation provides a complementary biological route for methane mitigation. Sewer-derived aerobic methanotrophs can enhance gaseous CH4 removal (Yuan et al., 2025), while nitrate dosing can stimulate nitrite-dependent anaerobic methane oxidation, providing an additional methane sink under anoxic conditions (Zuo Z. Q. et al., 2024). The practical effectiveness of these processes, however, depends on electron-acceptor availability, mass transfer, and microbial stability. Despite advances in characterizing sewer biofilm ecology and greenhouse gas emissions, critical knowledge gaps remain. First, the interactive effects of hydraulic conditions, microbial community dynamics, and metabolic pathways on CH_4_ and N_2_O production and consumption remain insufficiently quantified. Second, existing control strategies are largely reactive and pollutant-specific, often overlooking potential environmental trade-offs between different greenhouse gases. To date, no unified framework exists that integrates hydraulic reliability, biofilm-mediated biogeochemical processes, and multi-gas mitigation within a single management paradigm. Addressing these gaps is essential to reconciling urban drainage functionality with climate change mitigation goals.

This review critically synthesizes current knowledge on biofilm-mediated greenhouse gas emissions in urban sewer networks and the strategies available for their mitigation. The formation mechanisms, structural characteristics, and functional roles of sewer biofilms are characterized. Furthermore, the generation, microbial consumption, and emission pathways of CH_4_ and N_2_O are elucidated, with particular attention to methane oxidation mediated by methanotrophs and the accounting boundaries of greenhouse gas emissions. Subsequently, a critical synthesis of existing physical, chemical, and biological control strategies is provided, evaluating their underlying principles, effectiveness, and inherent limitations. Finally, priority future directions are identified, including multi-gas synergistic control technologies, precision regulation strategies, and integrated low-carbon management frameworks adopting a holistic “sewer network–wastewater treatment plant” system perspective. By addressing these dimensions, this review aims to advance the fundamental understanding of sewer biofilm processes and to inform the transition toward greener, low-carbon urban drainage infrastructure.

## 2 Sewer biofilm formation mechanisms

The biofilm on the inner walls of urban sewer networks is highly dynamic (Figure 1), manifesting as a continuous cyclic process of attachment, maturation, detachment, and re-diffusion (Sauer et al., 2022). The formation of the biofilm begins with the reversible contact of microorganisms following the formation of a conditioning membrane. This process then progresses to irreversible adhesion under the induction of surface sensing signals (Ren et al., 2024). Notably, surface structures such as flagella play a central role in this critical transition: they regulate intracellular second messengers [e.g., c-di-GMP cyclic diguanylate monophosphate (c-di-GMP) and sscyclic adenosine monophosphate (cAMP)] through signaling pathways upon surface contact (Webster et al., 2021). This regulation inhibits bacterial motility, promotes the early synthesis of extracellular polymers (EPS), and ultimately ensures stable microbial colonization on pipe walls (Zan et al., 2021).

**Figure 1:**
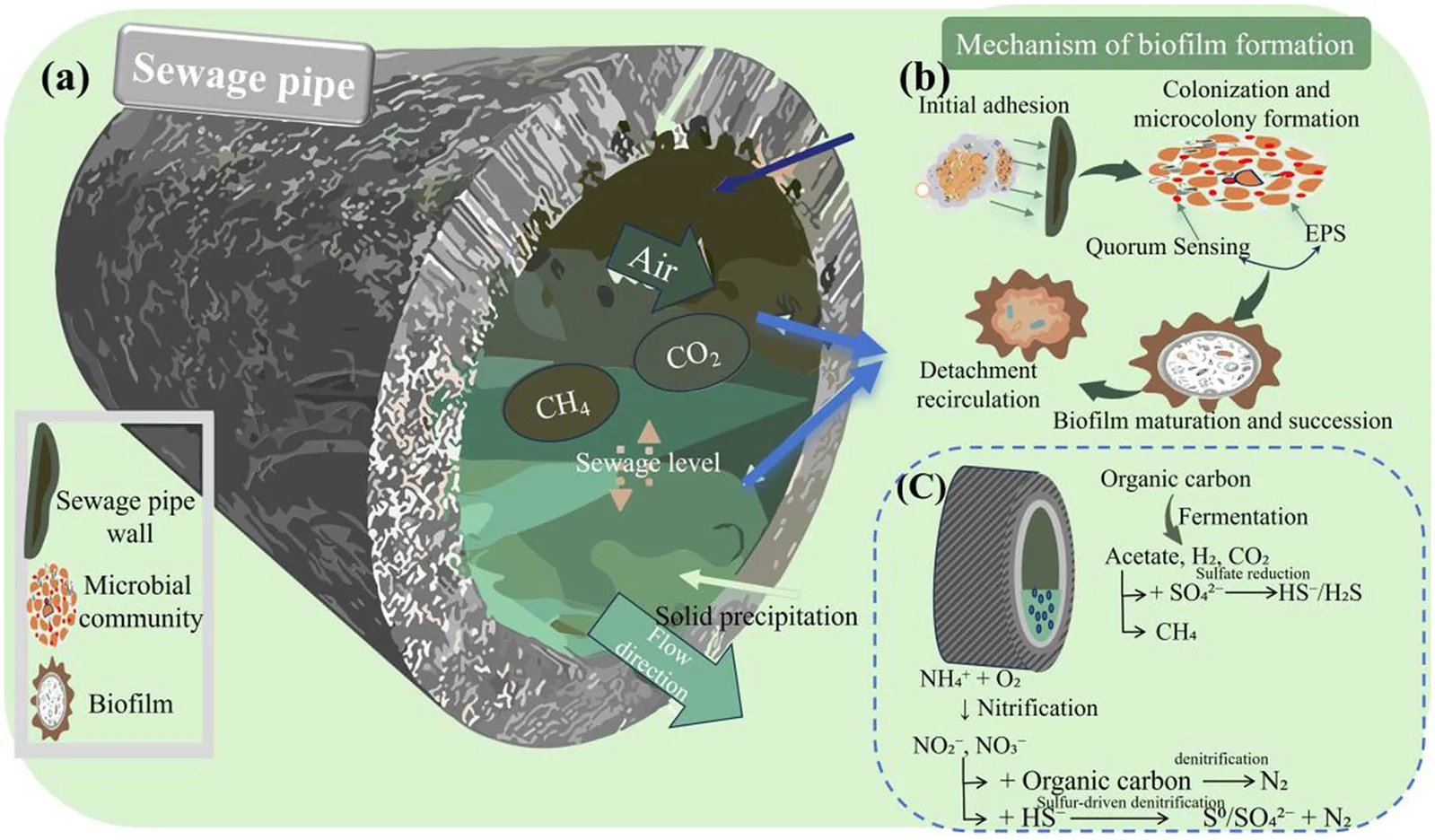
Mechanisms governing biofilm formation, microbially mediated greenhouse gas emissions, and pipe corrosion in urban sewer systems. **(a)** Schematic of the sewer network; **(b)** Dynamics of biofilm development; **(c)** Stratified microbial metabolism and associated corrosion.

As microbial clusters proliferate and accumulate, the biofilm expands from a two-dimensional plane into a complex three-dimensional system (Zheng et al., 2020). This network structure is supported by the matrix and characterized by distinct layers, pores, and channels (Figure 1). Because of limited material diffusion, this heterogeneous structure results in pronounced gradients of physical, chemical, and biological microenvironments within the biofilm (Böhning et al., 2024). In the mature stage, the combined effects of external hydraulic shear forces and internal matrix enzymolysis can lead to three distinct outcomes: localized erosion, block-like detachment of the biofilm as a whole, or the re-diffusion of disseminated cells (Zan et al., 2021). These processes promote some bacteria to revert to a planktonic state, allowing them to migrate to new sites for further colonization. Therefore, detachment is not just about biofilm disintegration; it is a crucial intrinsic mechanism for self-renewal, spatial expansion, and sustaining the life cycle of the biofilm.

In urban sewer networks, the close coupling of structure and function is closely linked to continuous material exchange between the biofilm, sewage, bottom sediments, and pipe wall surfaces (Tang et al., 2024). Consequently, the biofilm serves as both a key carrier for microbial community succession and an important reaction interface for biogeochemical processes like sulfate reduction and methane production within the pipeline system (Wu et al., 2023). At the functional level, material diffusion limitations within the biofilm create oxygen and redox gradients from its outer layers to interior regions. The exterior is conducive to aerobic or facultative metabolic processes, while deeper layers evolve into anoxic or strictly anaerobic microenvironments, fostering anaerobic activities such as fermentation, sulfate reduction, and methane production (Augustyniak et al., 2021; Hong et al., 2024). This spatial heterogeneity allows different microbial functional groups to coexist within the same biofilm, establishing a feedback network for substance and energy metabolism.

The formation of biofilms in urban sewer networks is influenced by three interrelated factors: hydraulic conditions, pipe surface properties, and particle deposition (Augustyniak et al., 2021; Hong et al., 2024). Hydraulic shear forces significantly impact the initial retention probability of microbial attachment and regulate both the structural density and shedding dynamics of the biofilm. Fluctuating shear conditions are particularly conducive to promoting spatial heterogeneity in biofilm growth (Tsagkari et al., 2022). Pipe surface characteristics, including roughness, pore structure, and chemical properties, directly determine microbial adhesion sites and local microenvironments. These factors exert selective pressures on biofilm community composition and metabolic functions, such as sulfur transformation processes (Hong et al., 2024; Kusum et al., 2020). Additionally, particle deposition in low-flow areas alters wall flow dynamics and mass transfer conditions, fostering stable anoxic microzones. The coupling between sedimentation and biofilm activity further amplifies spatial heterogeneity and enhances environmental risks associated with anaerobic metabolism, such as methane production (Tang et al., 2024; Yan et al., 2024). In conclusion, understanding and regulating pipeline biofilms necessitate a comprehensive analysis within this multifactorial coupled framework.

Quantitative observations underscore both the relevance and the uncertainty of this conceptual model. Sewer biofilms are commonly reported at approximately 1.0 mm thickness and may reach about 3.0 mm under some conditions (Zan et al., 2021), but neither biofilm thickness nor oxygen penetration depth is a universal property; both vary with flow, loading, surface characteristics, temperature, and sampling location. EPS composition, porosity, and channel structure regulate diffusion and detachment (Tsagkari et al., 2022), yet sewer-specific datasets that directly link EPS chemistry and microscale oxygen/redox profiles to CH_4_ and N_2_O fluxes remain scarce (Damgaard et al., 2001). Thus, redox stratification and the coexistence of functional guilds are well-supported, whereas predictive architectural thresholds for network-scale emissions remain an emerging research area.

## 3 Sewer biofilm-driven greenhouse gas generation and emission

### 3.1 Greenhouse gas emissions and explosion safety

Sediments and sewer biofilms will consume biodegradable organics in municipal wastewater and convert them into CH_4_ and CO_2_, resulting in carbon loss (Gao et al., 2023; Ye et al., 2024). Sediment type and hydraulic patterns influence methanogenic activity (Chen et al., 2021). CH_4_ emissions show strong spatial heterogeneity and intermittence; high emission points are often associated with vents, manholes, and other release nodes (Song et al., 2023). CH_4_ is the dominant non-CO_2_ greenhouse gas in sewer networks. Moreover, N_2_O can also be produced at local micro-oxic/anoxic interfaces (Joo et al., 2024; Yang et al., 2025). Mitigation should prioritize active sediment zones and major release nodes, while network-scale monitoring is needed to reduce uncertainties in CH_4_ and N_2_O accounting (Gao et al., 2023).

At the same time, sewer networks and manholes are confined spaces. Accumulation of flammable gases such as CH_4_ can lead to explosions when ignition sources are present; manhole structures can amplify blast consequences (Li et al., 2019). External natural gas leakage intruding into the sewer system is an additional important risk (Wang et al., 2021). Moreover, high water levels, poor ventilation, and low flow velocity conditions are particularly dangerous (Wang X. M. et al., 2025). Therefore, risk management requires a combination of continuous gas monitoring, forced ventilation, ignition source control, and entry permit systems.

Odor control should not be considered independently from methane safety. Sealing manhole covers may suppress fugitive odor locally, but can restrict natural ventilation and promote sewer gas accumulation or redistribution. In a full-scale gravity sewer, manhole cover opening altered airflow and transient CH_4_ and H_2_S peaks after intermittent pumping, highlighting the strong influence of cover configuration on local gas exposure (Matos et al., 2020). More importantly, a National Institute for Occupational Safety and Health (NIOSH) field investigation documented 57 manholes that had been sealed without adequate venting, with 1% methane subsequently detected in an adjacent mechanical room together with other sewer gases (Boudreau and Esswein, 2005). This case illustrates that unvented sealing can transfer an odor-management problem into a confined-space gas-accumulation hazard. Therefore, manhole sealing should be combined with engineered venting or gas treatment, pressure relief, and continuous CH_4_, H_2_S, and O_2_ monitoring rather than being implemented as an isolated odor-control measure.

### 3.2 Mechanisms underlying sewer biofilm-driven greenhouse gas generation and emission

The production and accumulation of CH_4_ in sewer networks occur primarily in anoxic or anaerobic microenvironments within biofilms and sediments. Organic matter is hydrolyzed and fermented to acetate, H_2_, and CO_2_ and is subsequently converted to CH_4_ through acetoclastic and hydrogenotrophic methanogenesis under syntrophic conditions (Joo et al., 2024). Substrate loading, hydraulic retention, temperature, and biofilm or sediment thickness regulate these reactions (Zan et al., 2020). Sulfate-reducing bacteria and methanogens compete for H_2_ and acetate, but spatial separation of electron acceptors and substrates allows competition and coexistence in different layers; the ${\text{COD/SO}}_{4}^{2-}$ ratio therefore redistributes carbon flow rather than acting as a simple on-off switch (Li et al., 2023). Pressurized sewers with limited reaeration and long residence times can accumulate dissolved CH_4_, whereas pumping stations, outlet manholes, drop structures, and other turbulent nodes can become atmospheric-release hotspots through pressure change and degassing (Figure 2) (Beelen and Parker, 2022).

**Figure 2:**
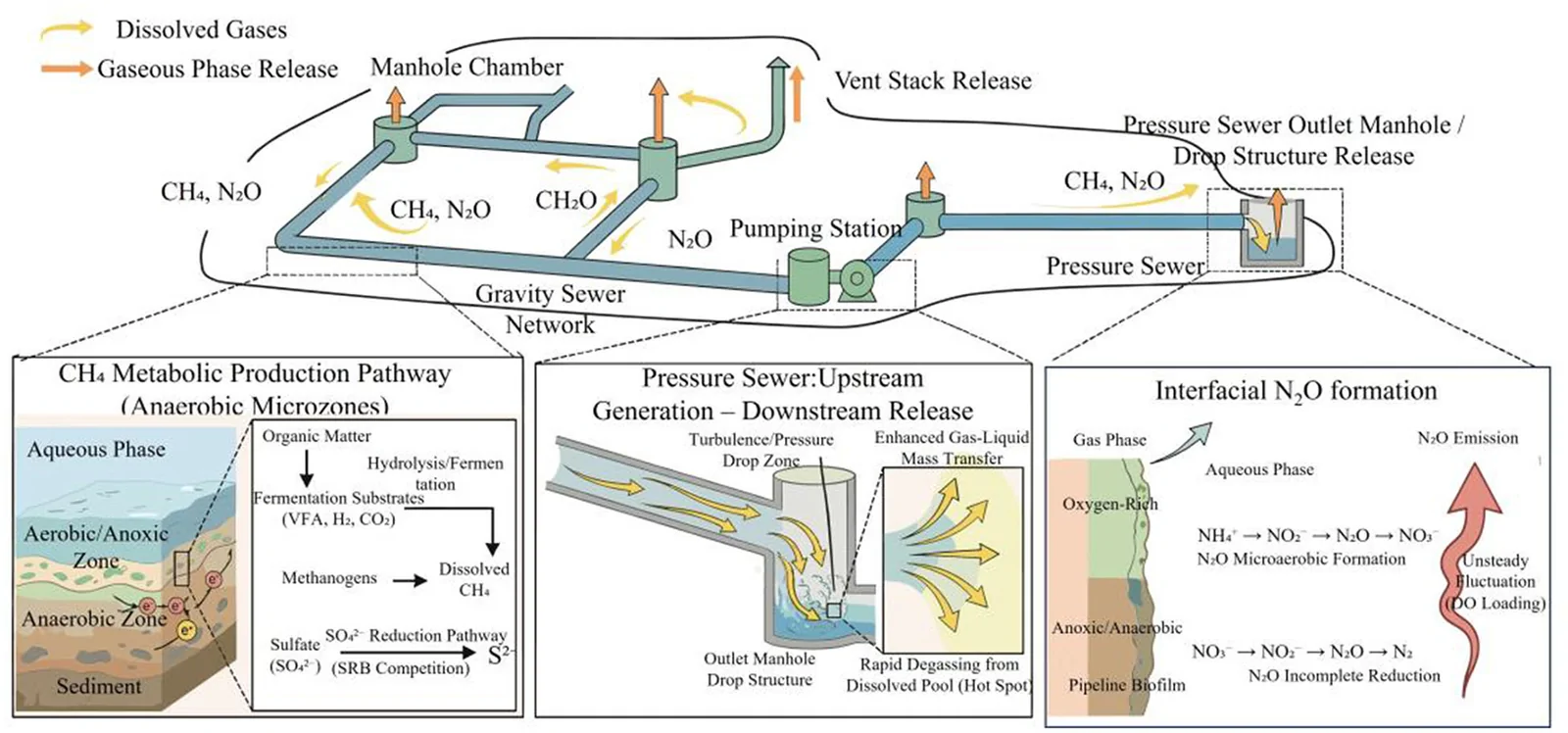
Mechanisms governing greenhouse gas emissions in municipal wastewater collection systems.

Methane oxidation is the counter-process that determines net CH_4_ release. Aerobic methanotrophs can consume CH_4_ at oxygenated biofilm-water or biofilm-gas interfaces, while nitrite-dependent anaerobic methane oxidation can couple CH_4_ consumption to nitrogen reduction under anoxic conditions. Nitrate or nitrite dosing may therefore suppress methanogenesis and promote methane consumption, but the balance depends on electron-acceptor delivery, mass transfer, residence time, and competition with sulfide oxidation and denitrification (Zuo Z. et al., 2024). Field-scale rates and persistence of these methane sinks remain less well-constrained than production, so gross production should not be equated with net emission.

Compared to CH_4_, N_2_O formation in sewer networks is more tightly coupled to interfacial microenvironments and operational dynamics. The stratified redox structure in biofilms and sediments—an oxic outer layer overlying an anoxic inner zone—promotes the micro-scale coexistence of nitrification and denitrification, creating hotspots for N_2_O production near interfaces (Yang et al., 2025). Under micro-aerobic or transient oxygen conditions, multiple pathways can be triggered: (i) hydroxylamine (NH_2_OH) oxidation—both enzymatic and abiotic—during nitrification, where accumulated NH_2_OH leads to N_2_O release (Olmo et al., 2026); (ii) nitrifier denitrification, in which ammonia-oxidizing bacteria reduce nitrite (${\text{NO}}_{2}^{-}$) to nitric oxide (NO) and then to N_2_O under oxygen limitation; and (iii) heterotrophic denitrification that becomes incomplete when the terminal reduction of N_2_O to N_2_ is restricted—a process governed by the activity of *nosZ*-carrying microorganisms and highly sensitive to trace oxygen, organic carbon availability, and environmental disturbances (Kim et al., 2022). Moreover, nitrate dosing, commonly applied for sulfide and corrosion control, reshapes the nitrogen oxide (NO_x_) pool and, under micro-aerobic conditions, may inadvertently intensify N_2_O accumulation by stimulating both heterotrophic and nitrifier denitrification pathways (via nitrite build-up). Concurrently, nitrate and its reduction intermediates competitively inhibit the activity of nitrous oxide reductase (N_2_OR), thereby suppressing the terminal reduction of N_2_O to N_2_ (Zuo Z. Q. et al., 2024). Hence, the dominant factor controlling N_2_O emissions in sewers is not simply the presence of a particular production route, but rather whether localized conditions drive the system into a “sensitive window”—characterized by enhanced intermediate buildup and impaired terminal reduction of N_2_O.

## 4 Factors influencing non-CO_2_ greenhouse gas production in sewers

The production of non-CO_2_ greenhouse gases in sewer systems is affected by the actual operation of complex sewer systems, such as season, weather, instantaneous sewage flow, and sewage discharge sources. Clarifying the influencing factors of non-CO_2_ greenhouse gases in sewer systems is the primary prerequisite for accurately accounting, predicting and controlling non-CO_2_ greenhouse gas carbon emissions in sewer systems. Thus, we summarized and sorted out the key factors that significantly affected the production of CH_4_ and N_2_O in sewer systems, mainly including sewage properties, external conditions and biofilm properties.

### 4.1 Sewage properties

The substrate concentration and composition in sewage are the key factors that determine the non-CO_2_ greenhouse gas production in sewer systems. The production of CH_4_ is based on organic matter in wastewater, and requires hydrolyzed fermentation, syntrophic production, homoacetogenesis, and methanogenesis. Within a certain concentration range, a higher organic matter concentration and a higher biochemical potential lead to a higher CH_4_ yield. The production of N_2_O is mainly based on inorganic nitrogen (e.g., ${\text{NH}}_{4}^{+}$, ${\text{NO}}_{2}^{-}$, ${\text{NO}}_{3}^{-}$) as the substrate. A higher concentration of inorganic nitrogen results in a higher N_2_O yield. Many nitrogen-containing organic substances will also generate inorganic nitrogen through hydrolysis, and thereby provide the substrate for N_2_O generation. However, when high-concentration industrial organic wastewater and kitchen wastewater are mixed in sewer systems, excessive substrate concentration in the sewage will enhance biological inhibition in short time and promote the accumulation of intermediate products such as organic acids, which may inhibit the production of CH_4_ and N_2_O within a certain time range (Wunderlin et al., 2012).

Dissolved oxygen (DO) is an important factor affecting the production of non-CO_2_ greenhouse gases in sewer systems. Most sewers have anaerobic and anoxic environments inside, but in some gravity sewers, sewage flowing through segment boundaries in different sewer diameters or different structures will cause the rise of DO due to water drops, which may lead to the coexistence of local aerobic and anaerobic or anoxic environments (Zuo et al., 2021). Excessively high DO will significantly reduce the activity of obligate methanogenic archaea (MAs), inhibiting the production of CH_4_. Generally, the DO concentration in pressure sewers is less than 0.1 mg/L, while the DO concentration in gravity sewers ranges from 0.1 to 0.4 mg/L, so the CH_4_ yield in pressure sewers is higher than that in gravity sewers (Li et al., 2023; Liu et al., 2015). Local DO concentration will affect NH_2_OH oxidation, ammonia-oxidizing bacteria (AOB) denitrification and heterotrophic denitrifying bacteria (HDN) reactions, and thus affect the yield of N_2_O. Under sufficient DO conditions, the local aerobic environment mainly undergoes NH_2_OH oxidation reaction with O_2_ as the main electron acceptor, which is conducive to NH_2_OH oxidation to NO and finally to N_2_O. When DO is low, the AOB denitrification reaction occurs, and ${\text{NO}}_{2}^{-}$ is used as the electron acceptor and reduced to the by-product N_2_O. In HDN, the denitrification under complete anoxic/anaerobic conditions will cause N_2_O to be completely reduced to N_2_. However, during denitrification with a small amount of DO, the N_2_OR activity is inhibited by O_2_, and N_2_O cannot be completely reduced to N_2_, indirectly leading to N_2_O accumulation (Frutos et al., 2018). Specifically, during HDN, the production of N_2_O is negligible when the DO content is less than 0.1 mg/L, but when the DO content increases to 2.4 mg/L, denitrification is hindered, resulting in a 28-fold increase in N_2_O emission in a lab scale sewer reactor (Zhang et al., 2022).

The pH of sewage, and the ${\text{NO}}_{3}^{-}$ and ${\text{NO}}_{2}^{-}$ contents in sewage will also affect the generation and emission of CH_4_ and N_2_O. The neutral environment of sewage in sewer systems is usually suitable for the growth of MAs and denitrifying microorganisms, while fluctuations in water quality and the interfusion of other wastewater may cause the sewage to be weakly alkaline in short time, inhibiting MA activity, enhancing nitrous oxide reductase (N_2_OR) activity and reducing CH_4_ and N_2_O production. In extreme cases, the overly high ${\text{NO}}_{3}^{-}$ content [(${\text{NO}}_{3}^{-}$) > 15 mg-N/L] and ${\text{NO}}_{2}^{-}$ content [(${\text{NO}}_{2}^{-}$) > 0.09 mg-N/L] (Jiang et al., 2011) in sewage, while promoting N_2_O generation, will increase the oxido-reduction potential, damage sewer biofilms and inhibit CH_4_ production.

While the individual effects of substrate concentration, DO, and pH on greenhouse gas production have been extensively documented, several critical caveats warrant attention. First, the reported threshold values for inhibitory effects [e.g., (${\text{NO}}_{2}^{-}$) > 0.09 mg-N/L (Jiang et al., 2011)] are predominantly derived from controlled batch assays or laboratory-scale reactors, and their transferability to real sewer networks—where substrate concentrations exhibit high spatiotemporal heterogeneity—remains largely unverified (Yang et al., 2025). Second, the interplay among multiple factors is rarely addressed; for instance, the combined effect of low DO and high organic loading on N_2_O yield may be synergistic rather than additive, yet current literature provides no consensus on such interactions. Third, most studies report net production or emission without distinguishing between gross generation and simultaneous consumption (e.g., CH_4_ oxidation, N_2_O reduction), which may lead to misinterpretation of the true metabolic capacity of sewer biofilms (Yang et al., 2025; Zuo Z. Q. et al., 2024). Future research should prioritize multi–factor experimental designs and *in–situ* validation to move beyond single–factor, laboratory–constrained generalizations.

### 4.2 Properties of biofilms

The interaction between microorganisms in biofilms, and the biofilm thickness are important factors affecting the generation and emission of non-CO_2_ greenhouse gases in the sewers. MAs and sulfate-reducing bacteria (SRBs) are functional microorganisms that produce CH_4_ and sulfides, respectively, and their coexistence in sewers will compete for electron donors (organic matter). Therefore, excessive SRB abundance in the biofilms of sewer systems will reduce CH_4_ yield (Guisasola et al., 2008). Anoxic and anaerobic biofilms generally have a layered structure in which SRBs dominate the outer layer and MAs dominate the inner layer, and the CH_4_ yield is negatively correlated with the penetration depth of sulfate (${\text{SO}}_{4}^{2-}$). Thus, a higher ${\text{SO}}_{4}^{2-}$ concentration in wastewater leads to a lower CH_4_ yield in biofilms (Sun et al., 2018). When the carbon-sulfur ratio (COD/S) in wastewater is high, the competitiveness of SRB is weak and the CH_4_ yield is less affected. Otherwise, the competitiveness of SRB is strong, which reduces MA abundance and thereby decreases the CH_4_ yield (Ai et al., 2019). The N_2_O yield in sewers is also related to the H_2_S generation from SRB reduction. N_2_OR is sensitive to H_2_S, and a high concentration of H_2_S will significantly inhibit N_2_OR activity, promoting N_2_O production (Pan et al., 2019, 2013). When the biofilms in sewers reach a steady state, the biofilm thickness is 1.0–3.0 mm (He et al., 2018). With the increasing thickness, the biomass rises, the DO levels in the biofilms and in the inner layer of sediments decrease significantly, and the proportion of anaerobic/anoxic regions increases. This is conducive to the enrichment of obligate and facultative anaerobic bacteria and CH_4_ production, but is not conducive to N_2_O production (Xu et al., 2017).

The description of biofilm thickness as a simple determinant of greenhouse gas production overlooks several important complexities. First, the reported “steady–state” thickness of 1.0–3.0 mm is derived from controlled laboratory systems; in real sewers, biofilm thickness varies significantly along pipe sections due to heterogeneous shear stress, sediment deposition, and intermittent flushing events (Xu et al., 2018), making it difficult to establish a universal thickness–emission relationship. Second, the internal stratification of biofilms is not static; it responds dynamically to diurnal flow variations, intermittent oxygenation, and substrate pulses, which may temporarily disrupt the outer SRB–dominated layer and expose inner methanogenic zones to inhibitory conditions—a phenomenon poorly captured by steady–state models (Xu et al., 2018). Third, the negative correlation between ${\text{SO}}_{4}^{2-}$ penetration and CH_4_ yield assumes a stable SRB/MA competitive balance, but this balance can be overturned by transient carbon surges or sudden changes in COD/S ratio (Ai et al., 2019; Li et al., 2023), leading to episodic CH_4_ pulses that are not predictable from average biofilm properties alone. Moreover, the reported H_2_S inhibition of N_2_OR activity is concentration–dependent, but the actual H_2_S concentration at the micro–scale within biofilms remains rarely measured, and the threshold for significant N_2_OR inhibition in sewer biofilms has not been systematically established. These gaps highlight the need for micro–scale profiling techniques (e.g., microsensors, stable–isotope tracing) to resolve the spatiotemporal heterogeneity of biofilm activity and its regulatory mechanisms.

### 4.3 External conditions

The hydraulic residence time (HRT), sewage flow rate and temperature of sewers significantly impact the yield of non-CO_2_ greenhouse gases in sewer systems. The CH_4_ concentration is approximately proportional to HRT. During the dry season or conventional flow, the sewage flow rate is small, the HRT is relatively long, and the dissolved CH_4_ concentration (accumulation) is high; during the rainy season or peak flow rate, the sewage flow rate is large, the HRT is relatively short, and CH_4_ accumulation is low (Guisasola et al., 2009; Short et al., 2017). Unlike the effect of HRT on CH_4_ in sewer systems, the dissolved N_2_O concentration in gravity sewers is approximately negatively correlated with HRT. This is because due to the increase of HRT, the DO concentration in biofilms further declines due to the consumption in microbial life activities, which in turn inhibits N_2_O production.

The sewage flow rate of sewers mainly affects non-CO_2_ greenhouse gas production by affecting the substrate mass transfer efficiency and the shear stress of the sewer wall. As the sewage flow rate rises, the mass transfer efficiency of intratubular oxygen (O_2_) is significantly improved, the efficiency of multiple oxygen-consuming biochemical reactions is enhanced and will aggravate DO consumption. Moreover, the proportion of anaerobic environment suitable for MA enrichment increases. As a result, the CH_4_ production (biological generation) in the sewers will increase with the rise of flow rate within a certain range (Xu et al., 2018). When the flow rate is higher than a certain threshold, the shear stress of the water flow impacting the sewer wall is significantly increased, the biofilm thickness is significantly reduced due to the erosion effect, and the sediments will also be disturbed by the high flow rate, resulting in a decrease in CH_4_ yield (Sun et al., 2015). However, it should be noted that this threshold is not a fixed value; its magnitude is highly dependent on sewer geometry and hydraulic conditions, including pipe diameter, surface roughness, bend curvature, sediment load, and flow regime (e.g., diurnal fluctuations vs. storm events). Furthermore, the impact of flow rate on N_2_O yield has been rarely reported, but it can be foreseen that the increase in flow rate will indirectly affect N_2_O production by improving the mass transfer efficiency of O_2_ and reducing biofilm thickness.

Temperature can affect the non-CO_2_ greenhouse gas production of sewer systems by affecting the metabolic rate and substrate supply of relevant microorganisms (Avery et al., 2003). Usually, the CH_4_ yield will also increase when the ambient temperature of sewer systems rises in summer. The actual data from the same segment of sewers on the Gold Coast of Australia show the average dissolved CH_4_ concentration in the sewers in summer is higher than that in winter (9.1 vs. 7.1 mg/L, average temperature 24.6 °C vs. 20.3 °C) (Liu et al., 2015). The optimal temperature of denitrifying bacteria (DNBs) is 30 °C−35 °C. The temperature fluctuations with seasons also affect the N_2_O yield by affecting DNB activity (Tan et al., 2020). For example, the low temperature in winter significantly reduces the reaction rate of N_2_O reduction to N_2_ during DNB denitrification, resulting in incomplete denitrification reaction and thereby increasing N_2_O yield (Chun et al., 2020).

Although the individual effects of HRT, flow velocity, and temperature have been repeatedly reported, the conclusions drawn from the literature are often conditional and sometimes contradictory, reflecting the complexity of real sewer systems (Sun et al., 2015). The positive correlation between HRT and dissolved CH_4_ concentration is well-established under steady flow, but during transient events (e.g., stormwater intrusion, pump station start–up), the relationship can be temporarily inverted due to enhanced stripping or dilution—a nuance that is rarely acknowledged in current accounting protocols (Song et al., 2023). Similarly, the reported positive effect of flow velocity on CH_4_ production holds only within a certain range; beyond a critical threshold, shear–induced biofilm sloughing and sediment resuspension reduce the active biomass, yet this threshold is system–specific and not readily generalizable. Temperature effects are similarly nuanced: while warmer temperatures generally stimulate methanogenesis, they also accelerate substrate depletion and increase maintenance energy requirements, which may limit the net CH_4_ yield under carbon–limited conditions—a feedback loop that is seldom captured in empirical correlations (Xu et al., 2018). For N_2_O, the reported negative correlation with HRT in gravity sewers is counter–intuitive and contradicts findings from some treatment reactor studies, suggesting that sewer–specific factors (e.g., biofilm stratification, sulfide interference) may override the general trend. This discrepancy underscores the need for integrated, sewer–specific kinetic models that incorporate the coupled effects of hydraulics, substrate availability, and biofilm architecture, rather than relying on generalized correlations imported from treatment reactor studies.

The effect of turbulence should be interpreted according to spatial scale. At drop structures, pumping-station outlets, bends, and manholes, local turbulence can enhance gas–liquid mass transfer and generate short-lived CH_4_ concentration or emission peaks. The associated safety risk is therefore primarily local and depends on confinement, ventilation, gas concentration, and the presence of ignition sources (Li et al., 2019; Matos et al., 2020). At the network scale, however, turbulence is generally secondary to microbial production, oxidation, dissolved and gaseous storage, transport, and final atmospheric release. Turbulence may change the location and timing of degassing without substantially reducing the network-integrated greenhouse gas burden. Consequently, a decrease in dissolved CH_4_ downstream of a turbulent node should not be interpreted as mitigation unless the stripped methane is captured or oxidized and the total phase-resolved flux is quantified. Sustained ventilation must also be distinguished from short-lived turbulence because oxygen delivery can alter redox conditions and microbial production. For example, upstream natural pulsed ventilation reduced methane concentrations by approximately 36%−42% in laboratory-scale gravity sewer systems, although field-scale persistence remains to be established (Gao et al., 2020).

## 5 Control strategies of greenhouse gas emissions

### 5.1 Reducing the formation of biofilms in sewer networks

Reducing biofilm formation in sewer networks requires targeting the fundamental conditions that facilitate its attachment, thickening, and anaerobization, rather than focusing solely on end-point removal of established biofilms (Zuo Z. et al., 2024). Easily biodegradable organics in wastewater, long-term accumulation on pipe walls and sediment surfaces, and hypoxic microzones formed under low-flow conditions jointly promote biofilm matrix development and the establishment of anaerobic metabolic zones, thereby increasing the potential for CH_4_ and sulfide generation (Shi et al., 2023). Therefore, biofilm control should primarily target the conditions that enable its formation, namely, reducing the supply of fermentable substrates, inhibiting sediment accumulation, and minimizing sustained hypoxic or anaerobic microenvironments (Figure 3).

**Figure 3:**
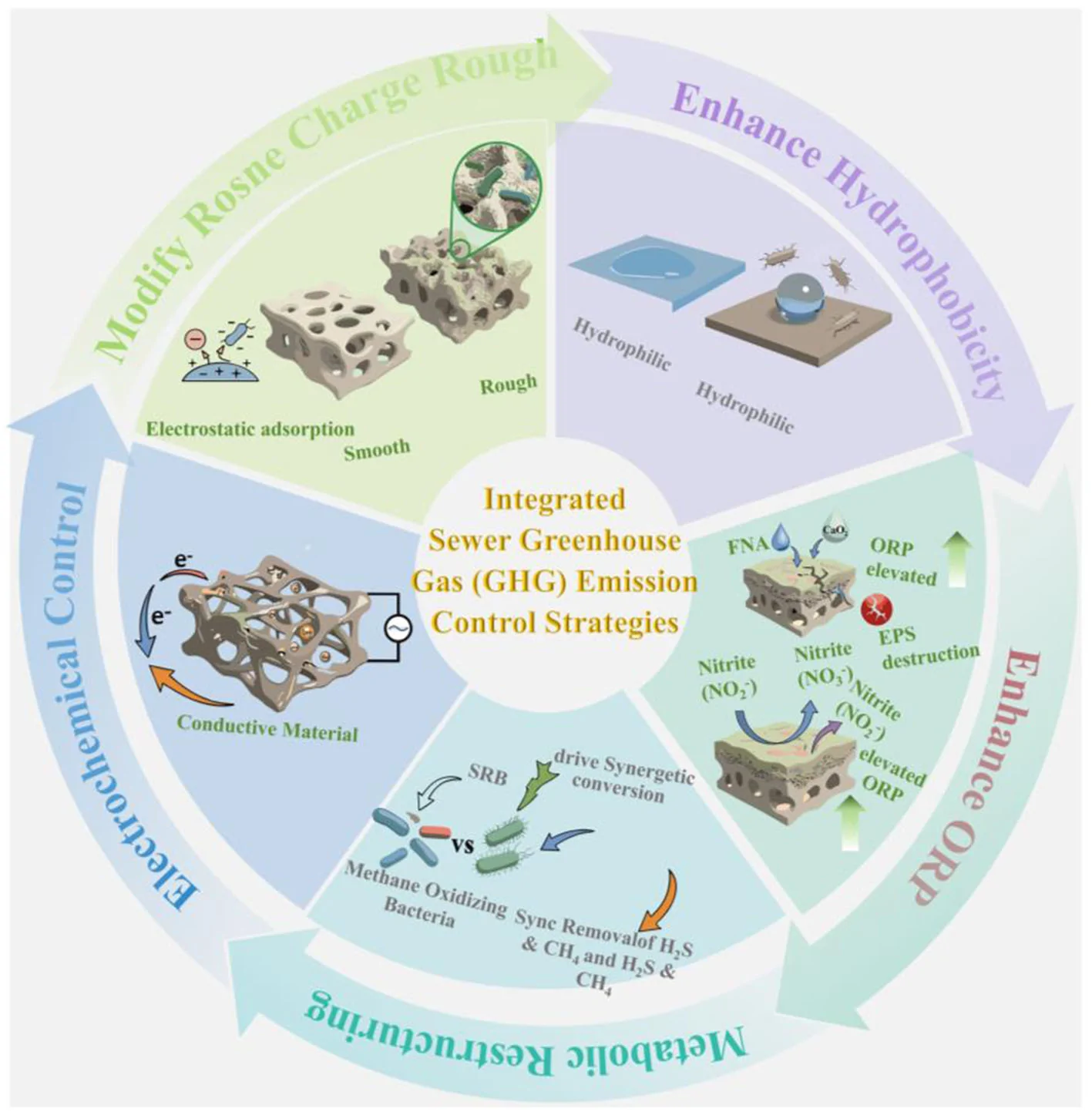
Synergistic strategies for greenhouse gas mitigation in urban sewer networks.

Existing control approaches can be broadly categorized into physical disruption and chemical intervention (Cen et al., 2023), sulfide control (Zhang et al., 2023), and nitrogen-based agents for sulfide and methane mitigation (Chen et al., 2025) (Figure 4). Ventilation, flushing, and physical disturbance targeting active sediment layers can destabilize attachment conditions on pipe walls and sediment surfaces, thereby limiting continuous biofilm thickening. Enhanced methods such as ultrasound have also been employed to reduce methane release potential in gravity sewers (Cen et al., 2025). Meanwhile, chemical agents such as CaO_2_, free nitrous acid (FNA), and Mg(OH)_2_ can delay the shift of biofilms toward sulfide- and methane-producing states by modulating local redox conditions, pH, and electron acceptor availability (Gao et al., 2023). Low-dose ferrate has further been shown to directly disrupt biofilm structure and matrix stability (Shi et al., 2025). However, the effectiveness of these measures remains influenced by hydraulic fluctuations, sediment re-accumulation, and pipe-section heterogeneity. Thus, the key lies not in short-term biofilm removal, but in sustainably undermining the interfacial conditions and microenvironmental foundations that support biofilm formation.

**Figure 4:**
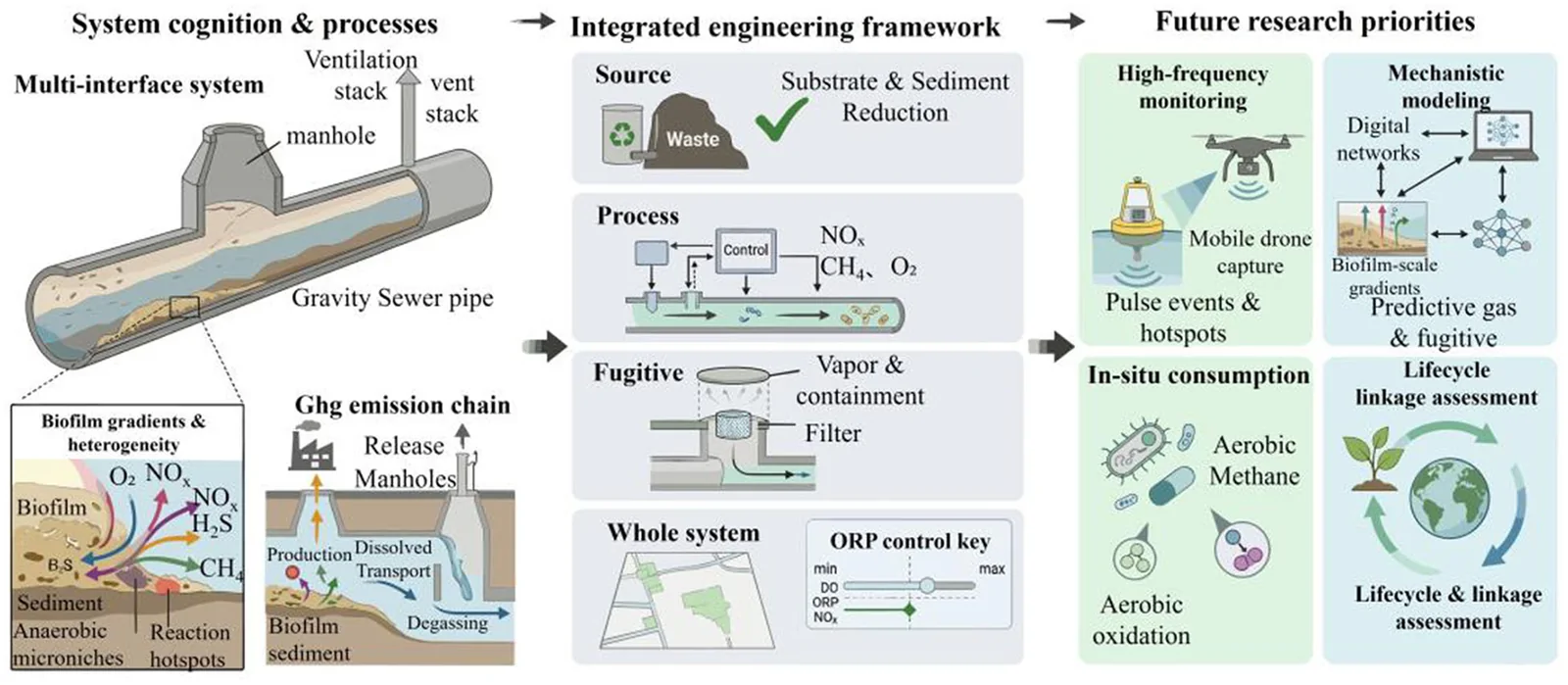
Approaches for control strategies of greenhouse gas emissions.

Despite the intuitive appeal of biofilm reduction strategies, their practical implementation faces substantial challenges that are often understated in the literature. Physical methods such as high-velocity flushing and mechanical cleaning require significant water consumption, operational downtime, and access to confined spaces. Chemical interventions, while effective under controlled conditions, may cause concrete corrosion, downstream microbial inhibition, and the formation of disinfection by-products; however, long-term field data on these collateral effects remain scarce. Moreover, the transient nature of biofilm removal—whereby disrupted biofilms can re–establish within days to weeks—suggests that intermittent interventions may yield only temporary benefits unless coupled with sustained hydraulic or chemical management (Ren et al., 2022). A further complication is the spatial heterogeneity of sewer networks: strategies validated in straight, accessible pipe sections may be ineffective in complex geometries (e.g., bends, drops, junctions) where flow conditions and biofilm characteristics differ markedly (Yan et al., 2023). The key knowledge gap is not whether biofilm reduction can reduce greenhouse gas emissions, but rather how to design adaptive, cost–effective, and site–responsive intervention schedules that account for the dynamic re–growth potential and spatial heterogeneity of sewer biofilms. A comparative assessment of these mitigation strategies, including their effectiveness, best-fit conditions, operational limitations, and environmental trade-offs, is summarized in Table 1.

**Table 1:** Comparative assessment of greenhouse-gas mitigation strategies for urban sewer networks.

Strategy	Effectiveness and life-cycle action	Best-fit conditions	Operational limitations	Environmental trade-offs	References
Flushing/sediment removal	Lowers active biomass, substrate inventory, and short-term production at targeted hotspots; durability requires repetition	Accessible gravity sewers and identified sediment hotspots	Transient effect; repeated operation; downstream solids handling	Resuspension and short-term stripping; loss of in-sewer transformation	Ren et al., 2022
Ventilation/forced aeration	Lower dissolved inventory and promote oxidation, but does not guarantee net emission reduction because gas may be transferred to the atmosphere	Confined hotspots where gas safety and capture can be managed	Energy demand; spatially uneven gas transfer	Atmospheric release can increase without capture or oxidation	Song et al., 2023; Yuan et al., 2025
Nitrate or nitrite dosing	Suppress methanogenesis and sulfide formation and support nitrite-dependent methane oxidation; performance is condition-sensitive	Pressurized or poorly aerated reaches with controllable dosing and mixing	Dose delivery, residual NO_x_, and continuous monitoring requirements	N_2_O accumulation under micro-aerobic conditions or incomplete denitrification	Zuo Z. Q. et al., 2024
Iron salts/oxidants/alkaline agents	Suppress biofilm activity, alter redox or pH, or disrupt EPS; reported effects are agent- and site-specific	Localized corrosion or sulfide/CH_4_ hotspots	Chemical consumption; recovery after dosing; material compatibility	Sludge generation, downstream chemistry, and non-target microbial effects	Guo et al., 2023; Yan et al., 2020
Bioaugmentation/methanotroph promotion	Potentially promotes CH_4_ consumption, but evidence remains mainly laboratory or pilot scale and long-term field effectiveness is uncertain	Locations with suitable O_2_ or alternative electron acceptors and adequate retention	Limited field-scale evidence; ecological stability	Competition for electron acceptors and uncertain persistence	Qi et al., 2026
Hydraulic and feedback control	Can reduce hotspot duration and target interventions; effectiveness depends on sensor coverage and model validity	Instrumented systems with controllable pumping, flushing, or dosing	Sensor coverage, model transferability, capital and maintenance needs	Energy use and possible redistribution of emissions to other nodes	Liang et al., 2024; Liu et al., 2016

### 5.2 Operational and hydraulic management

Sewer operation provides an additional mitigation opportunity by modifying hydraulic retention, oxygen transfer, and the duration of anaerobic exposure. Field measurements have shown that methane formation in rising mains increases with wastewater hydraulic retention time (Guisasola et al., 2008). More frequent pumping, lower wet-well operating levels, or coordinated pump scheduling may therefore reduce methane production when these measures shorten the combined retention time in the wet well and rising main. Network-scale coordination of pumping operations is technically feasible, as demonstrated by event-driven model predictive control developed for sulfide mitigation (Liu et al., 2016). However, the methane benefit of such control should not be assumed automatically because pump starts can also generate pressure waves, turbulent stripping, and short-lived gas peaks at downstream release nodes (Matos et al., 2020).

Persistent surcharge or submergence should likewise be avoided where operationally feasible. A field investigation found that pump-station operation submerged more than 1 km of an upstream trunk sewer, reducing flow velocity and increasing wastewater retention time (Guo et al., 2018). That study focused primarily on H_2_S rather than CH_4_; nevertheless, the same prolonged anaerobic retention is mechanistically consistent with enhanced methanogenesis in rising mains. Operational optimization should therefore balance shorter retention and improved ventilation against energy demand, pump wear, overflow risk, and the possibility of relocating emissions downstream. Its effectiveness should be verified using phase-resolved CH_4_ and N_2_O mass balances across the sewer network rather than concentrations measured at a single manhole.

### 5.3 Regulating the microbial community structure in sewer networks

Compared to reducing biofilm formation, regulating the microbial community structure offers a more direct approach to steering metabolic pathways within sewer networks (Figure 3). This strategy aims to reshape the electron acceptor landscape and competitive relationships among functional microorganisms, redirecting the system from fermentative, sulfate-reducing, and methanogenic processes toward lower-emission pathways such as methane oxidation and sulfide oxidation. Current research highlights the critical role of nitrogen compounds in this process. Nitrate and nitrite not only elevate the redox potential of the system, thereby suppressing sulfide and methane production, but also alter the coupling between carbon, nitrogen, and sulfur cycling, thereby restructuring the dominant functional groups in biofilms and sediments (Yuan et al., 2024). Consequently, the focus of community regulation should not be limited to “inhibiting a certain group of anaerobic bacteria,” but should shift toward redistributing electron flow and reshaping microbial competition patterns, allowing metabolic networks unfavorable to greenhouse gas accumulation to gradually become dominant.

At the practical level, nitrite-driven synergistic conversion processes have demonstrated strong potential. Studies show that nitrite can serve as a common electron acceptor, enabling simultaneous removal of sulfide and methane in sewers. This indicates that regulating electron acceptors can simultaneously influence multiple reductive pathways at the community level (Beelen and Parker, 2022; Shi et al., 2023). In addition, the use of aerobic methanotrophs derived from sewer environments can enhance gaseous CH_4_ removal, providing a new pathway from “suppressing generation” to “promoting consumption” (Zuo Z. Q. et al., 2024). Furthermore, conductive materials and electrochemical boundaries can alter the competitive relationships among electrogenic bacteria, methanogens, and sulfate-reducing bacteria, offering a basis for long-term, stable community restructuring (Zhang et al., 2025; Zhu et al., 2024). It should be noted, however, that while such strategies promise greater sustainability, their effectiveness depends heavily on DO, oxidation-reduction potential (ORP), and nitrogen oxides (NO_x_) delivery modes. Under nitrogen-based regulation, careful attention must be paid to avoiding the risk of N_2_O formation as a by-product. Therefore, community regulation should be integrated with operational boundary management to prevent CH_4_ reduction from being offset by the release of other high-global-warming-potential gases.

However, greenhouse gas mitigation in sewer networks cannot rely solely on a single chemical agent or a one-time physical intervention. Instead, it should advance concurrently on two fronts: controlling biofilm formation and regulating the microbial community. The former primarily targets attachment surfaces, EPS stability, and the formation of anaerobic micro-zones, while the latter focuses on electron acceptor supply, competition among functional microorganisms, and metabolic network reconstruction (Xu et al., 2025; Yan et al., 2025). These two approaches correspond, respectively, to reducing the formation of gas-producing habitats and shifting gas-producing metabolic directions. Only through coordinated implementation can stable emission reductions be achieved at the system scale. Future control strategies should further strengthen the integrated regulation of hydraulic conditions, interfacial micro-environments, and community ecology, and evaluate real emission reduction outcomes within a unified accounting framework and through on-site monitoring, thereby improving the comparability and scalability of strategies for practical engineering applications.

While microbial community regulation represents a conceptually appealing strategy, its translation from laboratory findings to field-scale application is constrained by several fundamental obstacles. First, the outcomes of community regulation are inherently stochastic and context-dependent; the same electron acceptor amendment (e.g., nitrate dosing) may yield divergent microbial responses in different sewer segments due to variations in baseline community composition, shear history, and substrate background, making predictive control exceptionally difficult (Chen Y. et al., 2024). Second, the long-term stability of the “restructured” community under realistic operational disturbances (e.g., flow surges, temperature drops, influent shocks) remains poorly characterized; most studies report short-term responses (days to weeks), and there is little evidence that the desired community shifts persist over seasonal or interannual timescales without repeated intervention. Third, the risk of unintended metabolic shifts—such as enhanced N_2_O production under nitrate-based regulation—is non–trivial and requires careful management of electron acceptor dosage, yet no operational guidelines currently exist for balancing CH_4_ suppression against N_2_O promotion in sewer networks (Zhang et al., 2022). Fourth, the scalability of bioaugmentation (e.g., introducing methanotrophs or competitive species) faces technical hurdles including strain viability during storage, uniform distribution throughout the network, and competition with established native communities (Yuan et al., 2025). Given these challenges, a prudent near–term approach would be to combine community regulation with continuous monitoring and feedback control (Liu et al., 2016), rather than relying on a fixed dosing regimen, and to adopt a system–wide mass balance framework to verify net greenhouse gas reduction rather than merely measuring CH_4_ or N_2_O in isolation (Yin et al., 2024).

## 6 Conclusions and future challenges

### 6.1 Conclusions

Urban sewer biofilms are dynamic reactive interfaces whose diffusion-limited redox gradients support methanogenesis, methane oxidation, nitrification, denitrification, and sulfate reduction. The existence of these coupled processes is well-established, but their relative rates and spatial organization under field conditions remain highly variable.

The climatic outcome cannot be inferred from production alone. Accounting must distinguish microbial production, aqueous and gaseous accumulation, network transport, and atmospheric release and must include gravity and pressure sewers, sediments and biofilms, manholes, vents, pumping stations, and dissolved-gas transfer to the wastewater treatment plant. Phase and location must be reported separately to prevent double counting and to identify emission transfer.

Mitigation strategies act by removing active biomass or substrate, changing redox conditions, promoting gas consumption, or transferring gas between phases. Their value should therefore be judged by net, multi-gas emission reduction and by effects on corrosion, safety, self-purification, downstream treatment, energy use, and chemical demand. Hotspot-targeted and feedback-enabled combinations are more defensible than blanket biofilm suppression or optimization for a single gas.

The scientific contribution of this review lies in integrating biofilm-scale microbial processes with network-scale greenhouse gas generation, transport, and emission. It further highlights the need to evaluate mitigation strategies from a multi-gas perspective, considering potential trade-offs among CH_4_, N_2_O, corrosion, safety, and operational demands. By extending the assessment boundary from individual sewer sections to the sewer–wastewater treatment plant (WWTP) continuum, this review provides a more comprehensive framework for identifying emission transfer and evaluating actual mitigation effectiveness. These insights provide a basis for developing more targeted and system-oriented strategies for low-carbon sewer management.

### 6.2 Outlook and research needs

Based on the foregoing synthesis, four research imperatives emerge as priorities for advancing the science and practice of sewer greenhouse gas management. First, monitoring and accounting protocols should harmonize system boundaries, dissolved and gaseous phases, units, temporal resolution, and treatment of sewer-to-WWTP transfer. Second, sewer-specific studies should quantify gross production and oxidation or reduction rates together, rather than inferring net emission from concentration alone. Third, experiments should resolve how geometry, hydraulics, EPS architecture, and carbon-nitrogen-sulfur competition create transferable thresholds or emission hotspots. Fourth, mitigation trials should report long-term, phase-resolved CH_4_ and N_2_O outcomes together with chemical, energy, corrosion, safety, and downstream-treatment consequences.

To mechanistically bridge biofilm-scale processes and network-scale fluxes, the integration of multi-omics profiling, microsensor measurements, stable-isotope tracing, and dynamic modeling holds considerable promise. Nevertheless, these advanced tools must be rigorously validated against field observations and explicit mass balances to ensure their predictive reliability. Furthermore, evidence derived from wastewater treatment reactor studies should be explicitly designated as cross-system evidence and critically re-evaluated under sewer-specific hydrodynamic and redox conditions before being extrapolated to sewer networks.

Ultimately, the practical objective is to establish a continuous, integrated management framework for the sewer–WWTP continuum. Such a framework should leverage high-frequency monitoring data to inform the selection and adaptive adjustment of hotspot-specific interventions, while simultaneously safeguarding against unintended consequences—most notably, the risk that reductions in CH_4_ emissions may be offset by concomitant N_2_O generation, or that dissolved gases may be merely transferred downstream to points of subsequent release. Adopting this systems-level perspective is, in our view, indispensable for achieving reproducible, verifiable, and genuinely low-carbon urban drainage management.
